# Occurrence of *Escherichia coli* and *Staphylococcus aureus* in Traditional Meat‐Based Foods and Cheeses Produced in Calabria (Southern Italy)

**DOI:** 10.1155/ijm/9978007

**Published:** 2026-08-16

**Authors:** Giulia Mancuso, Andrea Mancusi, Maria Garzi Cosentino, Silvia Daniela Cello, Maria Carmela Malagrinò, Francesco Mungo, Pierpaolo Palermo, Maria Antonietta Casbarra, Irene Dini, Yolande Therese Proroga

**Affiliations:** ^1^ Experimental Zooprophylactic Institute of Southern Italy, Cosenza, Calabria, Italy; ^2^ Experimental Zooprophylactic Institute of Southern Italy, Napoli, Italy; ^3^ Department of Pharmacy, University of Naples Federico II, Napoli, Italy, unina.it

**Keywords:** antimicrobial resistance MRSA, *Escherichia coli*, food safety, *Staphylococcus aureus*, traditional foods

## Abstract

Antimicrobial resistance (AMR) in foodborne pathogens represents a major public health concern, particularly in traditional animal‐derived foods. This study investigated the occurrence of *β*‐glucuronidase–positive *Escherichia coli* and methicillin‐resistant *Staphylococcus aureus* (MRSA) in 121 meat products and 328 cheeses produced in the province of Cosenza (Calabria, Southern Italy). Microbiological analyses were performed according to ISO 16649‐2:2001 and ISO 6888‐2:2021, and isolates were biochemically confirmed using the Vitek 2 System. Thirty cheese samples exceeded regulatory limits for coagulase‐positive staphylococci, while 29 showed elevated *Escherichia coli* counts, likely associated with raw milk use and artisanal processing. Among meat products, only two fresh sausages tested positive for *Escherichia coli*, suggesting contamination linked to inadequate sanitation of casings. Statistical analyses revealed significantly higher positivity rates for both microorganisms in cheese compared with meat products. These findings highlight the need for improved hygiene practices, continuous monitoring, and targeted interventions to reduce microbial contamination in traditional foods. Enhanced surveillance is also essential to track the emergence and dissemination of MRSA in the food chain.

## 1. Introduction

Antimicrobial resistance (AMR) occurs when microorganisms, such as bacteria, viruses, fungi, and parasites, adapt to resist drugs that were previously effective against them [1]. This resistance causes higher mortality rates and more prolonged illnesses [2]. The resistance observed in the affected population can be passed even among different microbial species. Understanding the prevalence of antibiotic‐resistant bacteria in food production is crucial for assessing the potential transmission through the food chain and estimating the risk of human exposure to these resistant strains [3]. The World Health Organization (WHO) recognizes AMR as one of the foremost global public health challenges [4]. Without intervention, it is estimated that by the year 2050, AMR could result in approximately 10 million fatalities annually. The economic ramifications are also considerable, with a potential annual decrease in global gross domestic product (GDP) of $3.4 trillion annually, which could result in an additional 24 million individuals falling into extreme poverty [5]. Addressing AMR requires a unified, multi‐sectoral approach called the “One Health” strategy, which emphasizes the interconnectedness of human, animal, and environmental health [6–8]. This approach involves improving infection prevention and control measures, promoting judicious antimicrobial use, and investing in the research and development of innovative treatments. The rise of *Staphylococcus aureus* and *Escherichia coli*, and their AMR, represents a significant challenge to public health. *Staphylococcus aureus* is a Gram‐positive, spherical bacterium characterized by non‐motility and lack of spore formation, although certain strains may possess a capsule. The organism can penetrate the skin and internal organs, leading to severe health issues in both animals and humans, including acne, skin infections, osteomyelitis, respiratory infections, endocarditis, and septicemia [9]. MRSA can develop resistance to *β*‐lactam antibiotics and virtually any other antibiotics. *β*‐lactam antibiotics induce bacterial cell death by inhibiting the production of penicillin‐binding proteins (PBPs) and inactivating enzymes involved in peptidoglycan synthesis (transglycosylases, transpeptidases, and carboxypeptidases). *Staphylococcus aureus* can inactivate the *β*‐lactam antibiotic’s action, synthesizing PBP2A (PBP2 ^′^, PBP2a; PBP2A is an altered target that remains unaffected by *β*‐lactam antibiotics, thus preserving its function in cell wall synthesis) and *β*‐lactamases and mutating PBP genes [10]. Individuals colonized with MRSA (meaning the bacteria are present without eliciting a detectable immune response, cellular damage, or clinical symptoms) face an elevated risk of subsequent infections and serve as critical vectors for person‐to‐person transmission. Healthcare settings that accommodate individuals at higher risk of infection due to invasive procedures or compromised immune systems experience substantial antibiotic selection pressure. This environment can lead to the emergence of AMR in bacteria, compounded by the frequent interactions among individuals [11]. *Escherichia coli* is a Gram‐negative bacterium in the genus *Escherichia*. It plays a crucial role in the intestinal microbiota of various animal species, including humans, and serves as a bioindicator for hygiene and AMR. *Escherichia coli* is among the most prevalent foodborne pathogens; ingesting food contaminated with specific strains can lead to serious health issues, including respiratory diseases, urinary tract infections, and pneumonia. *Escherichia coli* can resist antibiotics by producing beta‐lactamases (AmpC *β*‐lactamases and extended‐spectrum *β*‐lactamases) that alter target proteins in the cell wall, decrease outer membrane permeability, and enhance expression of drug efflux pumps [12]. *Escherichia coli* has developed the ability to produce various *β*‐lactamases, with extended‐spectrum *β*‐lactamases (ESBLs) and AmpC *β*‐lactamases (AmpC *β*‐lactamases). These enzymes exhibit distinct hydrolytic activity profiles. ESBLs are typically encoded by plasmids, with TEM and SHV variants among the first to be identified. However, over the past two decades, CTX‐M‐type ESBLs have emerged as the most prevalent enzymes in human and animal populations [13]. Unlike the SHV and TEM variants, CTX‐M groups are believed to have originated from chromosomally encoded ESBL genes found in various *Kluyvera* species. AmpC was initially recognized as a chromosomally encoded enzyme, although plasmid‐mediated resistance has also been observed. In *Escherichia coli*, the spread of the AmpC gene occurs through the horizontal transfer of plasmids, resulting in AmpC resistance in bacterial strains that previously had low or no expression of these genes [13]. Thus, methicillin‐resistant *Staphylococcus aureus* and *Escherichia coli* have emerged as significant zoonotic pathogens, posing considerable risks to public health and veterinary practices [14]. This work aimed to assess the prevalence of methicillin‐resistant *Staphylococcus aureus* and *β*‐glucuronidase‐positive *Escherichia coli* in Calabrian cheeses and meat products, providing valuable information on the presence of antimicrobial‐resistant strains for epidemiological studies. The enzyme *β*‐D‐glucuronidase is produced by 94%–96% of *Escherichia coli* strains, making its detection a valuable indicator of species identification [15]. Additionally, it is important to note that atypical STEC O157:H7 strains that display a *β*‐glucuronidase–positive phenotype (GP STEC O157:H7) have been infrequently isolated from humans; the research predominantly ruled out the likelihood of isolating this particular pathotype [16].

## 2. Materials and Methods

### 2.1. Sample Material

Four hundred and twenty‐eight (121 meat‐derived products and 328 dairy products) were examined, all sourced from local producers in Cosenza province.

#### 2.1.1. Meat‐Derived Products

Table 1 refers to the meat products tested.

**Table 1:** Categories of meat samples examined.

Categories	Number of samples
Neckpiece	1
Jowls	1
Nduja	11
Bacon	5
Salami	2
Sweet sausage	18
Fresh sausage	16
Spicy sausage	7
Cured sausage	23
Sweet cured sausage	1
Sopressata	21
Spicy sopressata	4
Total	110

The samples were grouped into three categories to improve the statistical analysis of the data (Table 2):

1. Calabrian sausage: includes sweet and spicy preparations (i.e., with sweet or hot chili pepper, respectively).
2. Calabrian sopressata: white or sweet, based on the absence/presence of chili pepper.
3. Other meat derivatives in which they are inserted: salami, nduja, bacon, and neckpiece.

**Table 2:** Distribution of meat samples by category and year.

Categories	2019	2020	2021	2022	Total
Calabrian sausage	11	18	30	6	65
Calabrian sopressata	6	11	4	4	25
Other meat derivatives	6	9	2	3	20
Total	23	38	36	13	110

#### 2.1.2. Milk‐Derived Products

Table 3 refers to the milk‐derived products tested.

**Table 3:** Categories of dairy samples examined.

Categories	Number of samples
Butirro	5
Caciocavallo	88
Basket cheese	18
Goat	11
Semi‐mature cheese	5
Cheese	2
Buffalo’s cheese	2
Mixed beef‐sheep‐goat’s cheese	1
Sheep‐goat’s cheese	28
Sheep’s chees	21
Freshly stretched curd cheese	5
Mozzarella	14
First salt	5
Provola	106
Ricotta	6
Scamorza	4
Thin slices	1
Spreadable cheese	1
Stracchino	5
Total	328

The samples were classified into three categories to enhance the statistical analysis of the data based on their production process and/or sample size:

1. Stretched curd cheeses made from bovine milk include provola, caciocavallo, mozzarella, and butirro (a specialty consisting of a stick of butter wrapped in stretched curd cheese).
2. Sheep and goat cheeses include both mixed‐milk and single‐species varieties.
3. Other types of cheese, such as buffalo cheeses, canestrato cheeses, thin cheeses, primo sale, and various kinds of spreadable cheeses, are considered (Table 4).

**Table 4:** Categories of cheese samples examined.

Categories	2019	2020	2021	2022	Total
Stretched curd cheeses made from bovine milk	87	73	59	2	221
Sheep and goat cheeses	31	17	16	1	65
Other types of cheese	16	10	11	5	42
Total	134	100	86	8	328

### 2.2. Isolation and Identification of *Escherichia coli* and *Staphylococcus aureus*

Bacterial colonies isolated during testing that exhibited the growth characteristics of the target organisms were further isolated and confirmed biochemically using the miniaturized Vitek 2 System (BioMérieux, Marcy l’Etoile, France). Reference strains used for testing bacterial identification were the following: *Staphylococcus aureus* ATCC 25923 (Microbiologics, St Cloud, United States); *Yersinia enterocolitica* ATCC 9610 (Thermo Scientific, Waltham, United States)*; Escherichia coli* NCTC 13216 (FEPTU, London, United Kingdom); *Escherichia coli* ATCC 25922 (Thermo Scientific, Waltham, United States), and *Listeria ivanovii* ATCC 19119 **(**Microbiologics, St Cloud, United States). These isolates were cultivated in 10 mL brain‐heart infusion broth (Biolife, Milan, Italy).

The resulting broth cultures were then aliquoted into cryo‐vials, each containing 2 mL of broth culture and 0.5 mL of sterile glycerol (Carlo Erba Reagents s.r.l., Cornaredo, Italy), and subsequently cryopreserved at −20°C in a specialized freezer. For each bacterial isolate, multiple aliquots were prepared for freezing to ensure the future recovery of the strain for antibiogram testing and any additional investigations deemed necessary. *Escherichia coli* *β*‐glucuronidase was detected according to ISO 16649‐2:2001 [17].

Each bacterial strain was assigned a designation (e.g., *Escherichia coli*) along with a unique numerical protocol, linked to it through the institution’s SIGLA information system, which connects back to the original matrix. In the enumeration of positive *Escherichia coli* *β*‐glucuronidase (UNI EN ISO 16649‐2: 2001) [17], graduated dilutions were prepared up to 10^−3^. Each dilution was plated by inclusion using Tryptone‐bile‐glucuronic medium Agar (Biolife, Milan, Italy), and incubated at 44°C ±1°C for 24 h. The colonies exhibiting the characteristic aqua‐green coloration were counted over two consecutive days.

Coagulase‐positive *Staphylococc*i, including *Staphylococcus aureus*, were detected using ISO 6888‐2 [18]. The tests were conducted on dilutions up to 10^−3^. Each dilution was inoculated onto Baird‐Parker Agar (Microbiol, Uta (CA), Italy), which was supplemented with rabbit plasma and bovine fibrinogen (Biolife, Milan, Italy), and incubated at 37°C ±1°C for 24 h. Reference strains used for testing bacterial identification were as follows:

*Staphylococcus aureus* ATCC 25923 (Microbiologics, St Cloud, United States); *Staphylococcus epidermidis ATCC 12228* (Thermo Scientific, Waltham, United States); *Staphylococcus saprophyticus ATCC BAA750* (Biomerieux, Marcy‐l′Étoile, France); *Escherichia coli* ATCC 25922 (Thermo Scientific, Waltham, United States), *Escherichia coli* NCTC 13216 (FEPTU, London, United Kingdom); *Enterococcu faecalis ATCC 29212* (Thermo Scientific, Waltham, United States); *Citrobacter freundii* ATCC 8090 (Thermo Scientific, Waltham, United States); and *Pseudomonas aeruginosa* ATCC 28853 (Thermo Scientific, Waltham, United States). The resulting data were analyzed using the Microincert Maxi Rev. 3b software (Spolaor Dino, Vicenza, Italy), which provides numerical output rounded to the first two significant digits in accordance with UNI EN‐ISO 7218 [19]. Thirty Calabrian cheese samples exceeded coagulase‐positive *Staphylococci* limits. Thirteen string cheese samples and 16 canestrati and pecorino samples surpassed the *Escherichia coli* *β*‐glucuronidase limit of 3000 CFU/g.

### 2.3. AMR

Antimicrobial susceptibility testing (AST) was performed on isolated strains of *Staphylococci* and *Escherichia coli* using the Vitek 2 System (BioMérieux, Marcy‐l’Étoile, France) according to the manufacturer’s guidelines. The testing was performed using standardized AST‐GP and AST‐GN cards for Gram‐positive and Gram‐negative bacteria. Each strain was tested against a panel of antibiotics commonly used in medicine. Minimum inhibitory concentrations (MICs) were interpreted according to the Clinical and Laboratory Standards Institute (CLSI) breakpoints [20–22]. For *Staphylococci aureus*, the antibiotics tested included benzylpenicillin, clindamycin, daptomycin, linezolid, oxacillin, rifampicin, teicoplanin, tetracycline, vancomycin, gentamicin, levofloxacin, tigecycline, and trimethoprim/sulfamethoxazole. Methicillin resistance was evaluated by oxacillin and cefoxitin susceptibility testing. For *Escherichia coli*, the panel included amikacin, gentamicin, nitrofurantoin, ciprofloxacin, tigecycline, meropenem, ertapenem, piperacillin/tazobactam, cefoxitin, cefotaxime, ceftazidime, and cefepime. Resistance to *β*‐lactam antibiotics was closely monitored due to the potential presence of extended‐spectrum *β*‐lactamases (ESBLs) or AmpC *β*‐lactamases.

### 2.4. Statistical Analyses

Chi‐square tests were used to evaluate the association between product category (meat vs. cheese) and qualitative microbial positivity. The exact test was reported for 2 × 2 tables to provide a conservative estimate when cell counts were low or *p* values were close to the significance threshold. Tests were performed using SPSS Statistics (Version 29; IBM Analytics, Armonk, NY, United States). The likelihood ratio chi‐square was included as a robustness check. Statistical significance was defined as *p* < 0.05, but borderline results (0.05 ≤ *p* < 0.10) were interpreted cautiously and discussed in terms of biological plausibility rather than strict statistical significance.

### 2.5. AI‐Assisted Tool

Grammarly was employed to improve the clarity and readability of the English text.

## 3. Results

One hundred and ten samples from local cured meat factories produced according to the traditions of the Calabria region, and 328 kinds of cheese were analyzed.

### 3.1. Microbiological Count Results

About 13.1% of dairy products were positive for *Staphylococcus aureus*, and 25.30% were positive for *Escherichia coli*. About 1.82% of meat products were positive for *Staphylococcus aureus*, and 5.45% were positive for *Escherichia coli.* Microorganisms were exclusively identified in fresh products (Table 5).

**Table 5:** Positivity rates of *Staphylococcus aureus* and *Escherichia coli* in different product categories.

Product category	Samples	*Staphylococcus aureus* positive	%	*Escherichia coli* positive	%
Stretched curd cheeses made from bovine milk	221	20	9%	53	24%
Sheep and goat cheeses	65	14	22%	12	18%
Other types of cheese	42	10	24%	18	43%
Sausage	65	2	3%	8	12%
Sopressata	25	0	0%	0	0%
Other meat	20	0	0%	0	0%

The distribution of qualitative *Staphylococcus aureus* positivity differed between meat and cheese products, with positive evaluations being proportionally more frequent in cheese (13.4%) than in meat (6.4%) (Table 6).

The chi‐Square test indicated a statistically significant association between product type and *Staphylococcus aureus* positivity (*χ* ^2^ = 3.98, *p* = 0.046). Although the continuity‐corrected chi‐square and the two‐tailed Fisher’s exact test did not reach the 0.05 threshold, the one‐tailed Fisher test (*p* = 0.029) supported the directional hypothesis that cheese products exhibit higher positivity rates than meat products. Taken together, these results suggest a weak but consistent association, which is statistically modest but biologically plausible given the intrinsic characteristics of cheese matrices and their production processes (Table 7).

**Table 6:** Distribution of qualitative staff evaluations across product categories for *Staphylococcus aureus* positivity.

Product type	Result	Count	% within type	% within *Staphylococcus aureus* qualitative	Total
**Meat**	Negative (N)	103	93.6%	26.6%	23.5%
	Positive (P)	7	6.4%	13.7%	1.6%
	**Total**	**110**	**100.0.0%**	**—**	**25.1%**
**Cheese**	Negative (N)	284	86.6%	73.4%	64.8%
	Positive (P)	44	13.4%	86.3%	10.0%
	**Total**	**328**	**100.0%**	**—**	**74.9%**
**Total**	Negative (N)	387	88.4%	100.0%	88.4%
	Positive (P)	51	11.6%	100.0%	11.6%
	**Total**	**438**	**100.0%**	**100.0%**	**100.0%**

**Table 7:** Statistical association between product type and qualitative staff evaluation of *Staphylococcus aureus* positivity.

Test	Value	df	Asymptotic sig. (2‐sided)	Exact sig. (2‐sided)	Exact sig. (1‐sided)
Pearson Chi‐square	3.981^a^	1	0.046	—	—
Continuity correction	3.325	1	0.068	—	—
Likelihood ratio	4.457	1	0.035	—	—
Fisher’s exact test	—	—	—	0.058	0.029
Number of valid cases	438	—	—	—	—

^a^No cells (0.0%) have an expected count below 5. The minimum expected count is 12.81.

The distribution of qualitative *Escherichia coli* positivity differs markedly between meat and cheese products. Positive samples were substantially more frequent in cheese (25.3%) compared with meat (7.3%). Cheese accounted for the vast majority (91.2%) of *Escherichia coli*‐positive findings, whereas meat accounted for only 8.8%.

Negative results were more common overall but remained proportionally lower in cheese (74.7%) than in meat (92.7%), reflecting the higher contamination rate observed in cheese products.

These data indicate that cheese products exhibit a significantly higher proportion of qualitative *Escherichia coli* positivity than meat products, suggesting potential differences in processing, handling, or intrinsic product characteristics that may influence microbial contamination risk (Table 8).

**Table 8:** Distribution of qualitative staff evaluations across product categories for *Escherichia **col**i* positivity.

Product type	Result	Count	% within type	% within *Escherichia coli* qualitative	Total
Meat	Negative (N)	102	92.7%	29.4%	23.3%
	Positive (P)	8	7.3%	8.8%	1.8%
	**Total**	**110**	**100.0%**	**—**	**25.1%**
Cheese	Negative (N)	245	74.7%	70.6%	55.9%
	Positive (P)	83	25.3%	91.2%	18.9%
	**Total**	**328**	**100.0%**	**—**	**74.9%**
Total	Negative (N)	347	79.2%	100.0%	79.2%
	Positive (P)	91	20.8%	100.0%	20.8%
	**Total**	**438**	**100.0%**	**100.0%**	**100.0%**

The chi‐square analysis (Table 9) reveals a highly significant association between the two categorical variables under investigation. The Pearson chi‐square statistic is large (*χ* ^2^ = 16,273, df = 1) and *p* < 0.001, indicating that the observed distribution differs markedly from what would be expected under independence.

**Table 9:** Statistical association between product type and qualitative staff evaluation of *Escherichia coli* positivity.

Test	Value	df	Asymptotic sig. (2‐sided)
Pearson Chi‐square	16,273^a^	1	0.000
Continuity correction	15,196	1	0.000
Likelihood ratio	19,203	1	0.000
Fisher’s exact test	—	—	—
Number of valid cases	438	—	—

^a^No cells (0.0%) have an expected count below 5. The minimum expected count is 22.85.

The continuity‐corrected chi‐square test, which provides a more conservative estimate for 2 × 2 tables, also remains highly significant (*p* < 0.001), confirming the robustness of the association. The likelihood ratio test further supports this conclusion, yielding a similarly strong significance level (*p* < 0.001).

Fisher’s exact test (Table 9), appropriate for 2 × 2 tables and particularly useful when expected frequencies are low, also returns *p* < 0.001 for both the two‐sided and one‐sided tests. Although the expected cell counts were adequate for chi‐square approximation, Fisher’s test provides convergent evidence of a strong association. Taken together, these results demonstrate a highly significant relationship between the variables, with the direction and magnitude of the association consistent across all inferential tests. The absence of low expected cell counts further supports the validity of the chi‐square approximation.

The coagulase‐positive *Staphylococci* count and the positive *β*‐glucuronidase *Escherichia coli* count were reported in Table 10. The 95% confidence intervals for mean microbial counts showed non‐overlapping ranges between cheese and meat categories, indicating significantly higher contamination levels in cheese. The wide intervals observed in sheep and goat cheeses reflect greater variability in artisanal production practices, whereas the narrow intervals in meat products indicate more uniform microbiological profiles.

**Table 10:** AMS’ count according to the UNI EN ISO 6888‐2/2004‐2021 (*Staphylococci* count) [18] and UNI EN ISO 16649‐2:2001 (positive *β*‐glucuronidase *Escherichia coli* count) [17].

Product category	Mean (CFU/g)	Min.	Max	Standard deviation	Positive samples (%)	*n*	95% CI (lower–up)
*Staphylococcus aureus*							
Stretched curd cheeses made from bovine milk	380,942	20	2,400,000	718,024	20%	221	[283,660–478,224]
Sheep and goat cheeses	32,880	350	130,000	34,316	14%	65	[24,502–41,258]
Other types of cheese	34,147	170	83,000	36,371	10%	42	[22,084–46,210]
Calabrian sausage	1915	30	3800	2,666	2%	65	[1,266–2,564]
Calabrian sopressata	0	0	0	0	0%	25	0 (no variability)
Other meat	0	0	0	0	0%	20	0 (no variability)
*Escherichia coli* *β*‐Glucuronidase‐positive							
Stretched curd cheeses made from bovine milk	5157	20	140,000	19,595	53%	221	[2,554–7,760]
Sheep and goat cheeses	180,878	700	1500,000	441,567	12%	65	[73,807–287,949]
Other types of cheese	29,064	200	150,000	40,869	18%	42	[16,650–41,478]
Sausage	1181	50	5000	1938	8%	65	[709–1,653]
Sopressata	0	0	0	0	0%	25	0 (no variability)
Other meat	0	0	0	0	0%	20	0 (no variability)

### 3.2. *Staphylococcus aureus* Resistance

**Figure 1:**
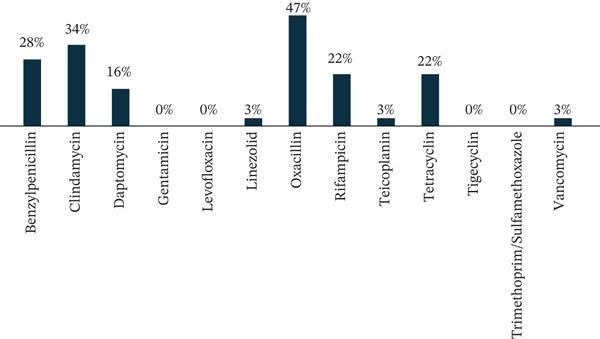
Distribution of the *Staphylococcus aureus’* resistance phenomenon.

Thirty‐two strains of *Staphylococcus aureus* were examined, revealing that nine exhibited resistance to benzylpenicillin, 11 to clindamycin, five to daptomycin, one to linezolid, 15 to oxacillin, seven to rifampicin, one to teicoplanin, seven to tetracyclines, and one to vancomycin (Figure 1). Resistance was not observed against gentamicin, levofloxacin, tigecycline, and trimethoprim/sulfamethoxazole.

**Figure 2:**
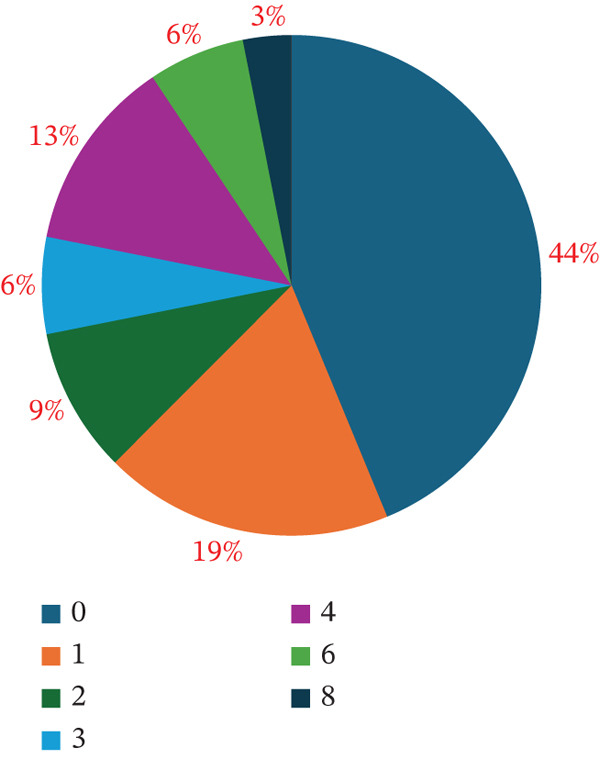
Antimicrobial resistance of *Staphylococcus aureus* strains. The legend shows the number of molecules to which the strains were resistant.

Twelve strains of *Staphylococcus aureus* (37.5%) demonstrated resistance to two or more molecules (up to eight): 9% were resistant to two antibiotics, 6% were resistant to three antibiotics, 13% were resistant to four antibiotics, 6% were resistant to six antibiotics, and 8% were resistant to eight antibiotics (Figure 2). Four *Staphylococcus aureus* strains exhibited intermediate sensitivity to ertapenem and resisted three antibiotics: piperacillin/tazobactam, cefoxitin, and ceftazidime. These strains were excluded from the evaluation of AMR because they were considered intrinsically less precise and more prone to statistical interpretation errors [20, 21].

### 3.3. *Escherichia coli* Resistance

**Figure 3:**
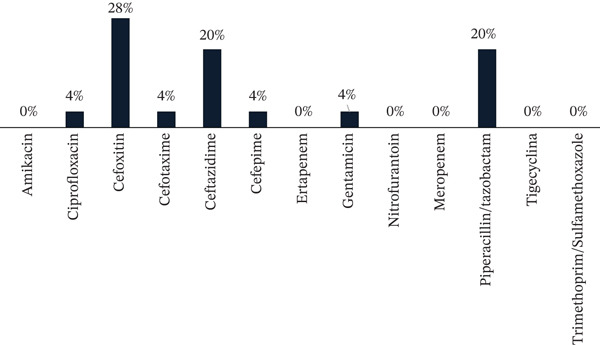
Distribution of the *Escherichia coli’s* resistance phenomenon.

**Figure 4:**
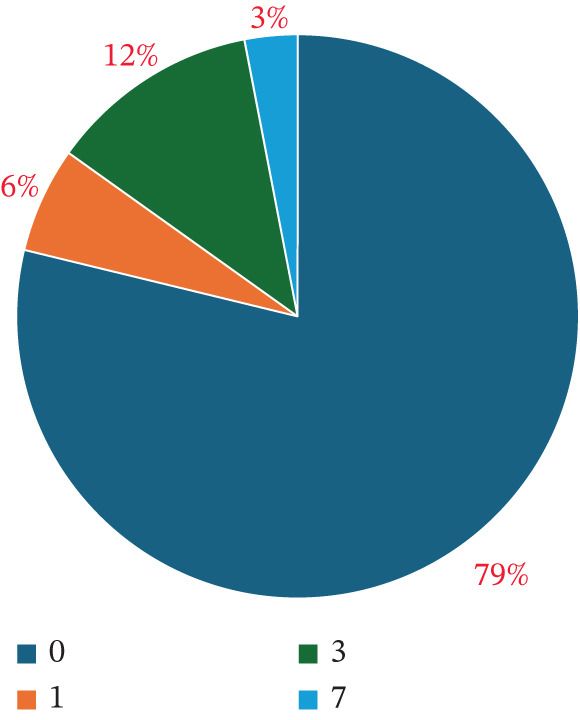
Antimicrobial resistance of *Escherichia coli* strains. Legend reports the number of molecules against which the strains were resistant.

Thirty‐two strains of *Escherichia coli*, isolated from milk products, were evaluated for susceptibility. Two strains resisted cefoxitin, while four demonstrated multi‐resistance to cefoxitin, ceftazidime, and piperacillin/tazobactam. No resistance was detected against cefoxitin, cefotaxime, cefepime, gentamicin, nitrofurantoin, meropenem, piperacillin/tazobactam, and tigecycline. Additionally, intermediate sensitivities were observed for specific agents: amikacin (one strain), cefepime (two strains), and ertapenem (Figures 3 and 4). These strains were excluded from the AMR analysis because intermediate susceptibility is considered less reliable and more prone to misclassification [20, 21]. Five strains from fresh sausage samples were evaluated. One strain showed multi‐resistance to seven antibiotics: piperacillin/tazobactam, cefoxitin, cefotaxime, ceftazidime, cefepime, gentamicin, and ciprofloxacin; the other strains did not show AMR.

Five strains of *Escherichia coli* (15.6%) demonstrated resistance to two or more antibiotics (up to 7): 12% were resistant to three antibiotics, 3% were resistant to seven antibiotics (Figure 4).

## 4. Discussion

This work evaluated the presence and AMR of S*taphylococcus aureus* and *Escherichia coli* *β*‐glucuronidase in Calabrian cheeses and meat products sourced from local producers in Cosenza province. Based on cross‐referenced data from the Chamber of Commerce of Cosenza (Movimprese reports) and an assessment of the local production system (Consortia and Trade Associations) [22], an average of 115 processing companies were active in the province of Cosenza during 2019–2021 for ATECO code 10.51 (Dairy Industry) [23], which includes enterprises whose primary activity is milk processing (excluding agricultural holdings processing only their own milk). During the study period, 328 cheese samples originating from 79 different production facilities were analyzed. For the same reference period, companies operating in the meat processing, preservation, and meat‐based product manufacturing sector (ATECO code 10.13) numbered an average of 82 in the province of Cosenza. In our study, 121 meat products from 41 distinct production facilities were examined. The analyses revealed that the established limit for coagulase‐positive *Staphylococci* was exceeded in 30 cheese samples. Additionally, 13 samples of string cheese exceeded the permissible limit for *Escherichia coli* *β*‐glucuronidase, while 16 samples from other categories, specifically canestrati and pecorino, exhibited *Escherichia coli* *β*‐glucuronidase levels exceeding 3000 CFU/g.

In the production of Calabrian stretched curd cheeses, the maturation process of the curd is deemed complete when it is suitable for stretching. Small samples of the pasta are periodically taken and immersed in nearly boiling water (70°C–90°C) to determine whether the pasta can be stretched into elastic, shiny, continuous, and resilient fibers. The formed shapes are then immersed in cooling water before being placed in brine, with the salting time varying with the cheese’s weight [24]. Variations in brine also exist, particularly concerning salt concentration and temperature. The temperature of the water during the spinning process and its duration are critical, with numerous subjective variations noted in this area.

For caciocavallo silano, specific regulations outline production standards for time and temperature, while the production of provola and other stretched‐curd cheeses relies on the individual cheesemaker’s expertise, resulting in distinct product characteristics. Current legislation (E.C. 2073/2005) [25], which establishes hygiene criteria for milk and its derivatives, sets the limit for coagulase‐positive *Staphylococcus aureus* and *Escherichia coli* at 1000 CFU/g. Considering the stages of the production process, the detection of *Escherichia coli* and coagulase‐positive *Staphylococci* in the examined products may result from improper handling, leading to secondary contamination, as well as from a significant bacterial load present in the raw materials, which creates an environment conducive to growth during the maturation phase, despite competition from lactic flora. Concerning meat products, the traditional cured sausage known as soppressata is made by fermenting shredded, salted raw meat, combined with shredded fat seasoned with various spices, and then encased in natural or artificial casings. In the province of Cosenza, there is a production specification for Soppressata di Calabria DOP [26]. Other varieties of cured meats are crafted according to age‐old recipes that differ among producers. The crafting of Calabrian cured meats prominently features the use of chili pepper alongside salt. Salt is crucial for dehydrating meat through an osmotic effect that draws water and enzymes out of the meat. Chili peppers are used in both sweet and spicy forms, and their antibacterial properties have been recognized for some time [27]. Regarding meat products, Regulation 2073/2005 does not impose specific limits for this category [28]. However, acceptable levels of *Escherichia coli* in products intended for cooking are < 1000 CFU/g, whereas in raw products they are < 100 CFU/g [29]. Concerning *Staphylococci,* a maximum value of ≤ 100 CFU/g is required, regardless of whether the products are intended for cooking or consumption raw [30]. Only two Calabrian meat products were positive for coagulase‐positive *Staphylococci*, both of which were fresh. One of these products exhibited a load result exceeding 1000 CFU/g, although it remained below the threshold that necessitates testing for *Staphylococcus aureus*. Finally, eight exhibited *Escherichia coli* *β*‐glucuronidase counts exceeding 10 CFU/g, with two exceeding 1000 CFU/g. All of these instances were associated with fresh sausages, suggesting that the contamination likely originated from the use of inadequately sanitized natural casings during sausage production [31]. It is possible that the seasoned Calabrian products, due to their production methods, do not provide a conducive environment for the proliferation of the two target strains studied. In Calabria, unlike other regions of Italy, fresh sausage is intended for consumption only after thorough cooking, which may reduce the bacterial load observed in these products. The surveillance data reviewed indicate that MRSA occurrence in pork‐derived fermented meats remains consistently low. These findings align with previous evidence showing that contamination primarily arises during slaughtering and handling rather than persisting in the final product. Technological hurdles inherent to fermentation and ripening, including acidification (pH < 5.2) and reduced water activity, can limit MRSA survival in ready‐to‐eat products [31, 32]. Likewise, the very low detection of MRSA in traditional cheeses indicates that dairy products do not constitute a major exposure route for consumers when appropriate hygiene measures are implemented. Although *Staphylococcus aureus* is frequently isolated from raw milk, pasteurization and the application of good manufacturing practices substantially reduce the associated risk, as consistently reported in EFSA surveillance assessments [32]. In contrast, ESBL‐producing Enterobacterales, particularly *Escherichia coli*, remain a significant concern in the food chain. Although several EU Member States have reported a decline in pork meat prevalence following reductions in antimicrobial use in livestock, ESBL genes remain detectable in raw meat intended for further processing [31]. This persistence highlights the need to monitor resistance determinants at the primary production level rather than focusing exclusively on finished products. Although pathogenic ESBL‐producing bacteria are only rarely isolated from these products, several studies have reported the presence of commensal and lactic acid bacteria carrying resistance determinants. Such microorganisms may facilitate the dissemination of ESBL genes through horizontal gene transfer, thereby acting as reservoirs within the food‐associated resistome [31, 33–34]. Our findings indicate that the principal risk associated with traditional fermented foods lies not in direct exposure to resistant pathogens, but in the potential for AMR genes to circulate along the food chain.

## 5. Conclusions

This study provides an updated assessment of the microbiological quality of traditional cheeses and meat products produced in the province of Cosenza (Italy). While most samples complied with current hygiene criteria, some raw‐milk cheeses exceeded regulatory limits for *Staphylococcus aureus* and *Escherichia coli* *β*‐glucuronidase, underscoring the importance of proper handling and hygiene during production. In contrast, cured meat products showed minimal contamination, likely due to processing conditions that inhibit bacterial growth. The very low MRSA detection rate confirms that, when good manufacturing practices are applied, these foods do not represent a major exposure route for consumers. However, the persistence of ESBL‐producing *Escherichia coli* and the potential role of commensal bacteria as reservoirs of resistance genes highlight the need for continued AMR surveillance along the food chain. This work presents some limitations. The study was restricted to a single geographical area, and although the sample size reflects the actual number of active processing facilities, the number of isolates available for AST was limited. Moreover, resistance was assessed solely by phenotypic methods, without molecular confirmation of resistance genes. Future studies should expand the geographical scope, increase sampling coverage, and incorporate molecular analyses to characterize resistance mechanisms and their dissemination.

## Author Contributions

Conceptualization, Giulia Mancuso, Andrea Mancusi, and Yolande Therese Proroga; formal analysis, Maria Garzi Cosentino, Andrea Mancusi, Silvia Daniela Cello, Maria Carmela Malagrinò, Pierpaolo Palermo, Francesco Mungo, Maria Antonietta Casbarra; resources, Yolande Therese Proroga; data curation, Giulia Mancuso, Andrea Mancusi, Yolande Therese Proroga, Irene Dini; writing – original draft preparation, Irene Dini; writing – review and editing, ID.

## Funding

This study was funded by the Ministry of Health, General Directorate of Veterinary Health and Veterinary Medicines as part of the current research projects in 2019 (IZSME 10/19RC). Open access publishing facilitated by Universita degli Studi di Napoli Federico II, as part of the Wiley ‐ CRUI‐CARE agreement.

## Disclosure

All authors read and approved the final manuscript.

## Ethics Statement

The authors have nothing to report.

## Conflicts of Interest

The authors declare no conflicts of interest.

## Data Availability

The data that support the findings of this study are available from the corresponding author upon reasonable request.
